# A Survey of Operative Techniques Used by Female Pelvic Medicine and Reconstructive Surgeons Performing Minimally Invasive Sacral Colpopexy

**DOI:** 10.7759/cureus.10931

**Published:** 2020-10-13

**Authors:** Alexandra Dubinskaya, Kaitlin Renkosiak, Jonathan P Shepherd

**Affiliations:** 1 Urology, Cedars Sinai Medical Center, Los Angeles, USA; 2 Obstetrics and Gynecology, St. Francis Medical Center, Hartford, USA; 3 Female Pelvic Medicine and Reconstructive Surgery, University of Connecticut School of Medicine, Hartford, USA

**Keywords:** minimally invasive surgery, sacrocolpopexy, pelvic organ prolapse

## Abstract

Objective

Assess variability of surgical technique for minimally invasive sacral colpopexy (MISC) among Female Pelvic Medicine and Reconstructive Surgery (FPMRS).

Methods

A voluntary anonymous questionnaire was given to the 2018 American Urogynecologic Society (AUGS) annual meeting attendees. Comparisons were made by age, gender, experience (years), practice setting, and U.S. region.

Results

There were 59 responses from 671 physician conference attendees. Most were male (64.4%), U.S. physicians (94.6%), completed Obstetrics and Gynecology residencies (91.5%), practicing in University settings (66.1%). The mean age was 47.4±8.6 years, practicing>15 years (47.5%). Predominant routes were 53.8% robotic, 42.2% laparoscopic, and 4.0% open. Surgeons used 3-4 ports (both 50.0%), with 0-degree (46.0%) or 0 and 30 degree laparoscopes (36%). For sacral mesh attachment, 83.1% used suture as opposed to tacking devices, most often Gortex (56.3%). Anterior (48.1%) and posterior (50.0%) vaginal attachment used 5-6 sutures.

Concomitant procedures included anterior repair (83.4% “not usually”/“not at all”), posterior repair/perineorrhaphy (77.8% “yes, often”/“yes, sometimes”), midurethral sling (42.6% “yes, often”/51.9% “yes, sometimes”), and hysteropexy (86.5% “not usually”/“not at all”). Post void residual (PVR) was assessed after surgery by 89.8%, 75.5% via retrograde fill voiding trial. Most patients were discharged post-operative day 1 (POD1) (47.6% AM, 29.1% PM) or day of surgery (15.2%).

Females more commonly performed hysteropexy (p=0.028) with no other significant differences by age, gender, experience, practice setting or region.

Conclusion

Most FPMRS surgeons perform MISC, equally robotic and laparoscopic. Concomitant posterior wall procedures and midurethral slings are common. Other than more hysteropexies performed by females, no other variables predicted technique variations, suggesting technique homogeneity.

## Introduction

The lifetime risk of a woman undergoing surgical repair of pelvic organ prolapse (POP) is greater than 12% [1]. Sacral colpopexy (SCP) is considered the gold standard procedure for apical prolapse repair. First described in 1962 by Lane, the abdominal SCP uses biological or synthetic material attached to the anterior longitudinal ligament of the sacrum to provide apical support [2]. Over the last several decades, SCP evolved from an open to a minimally invasive technique.

Current literature suggests that minimally invasive approaches decrease morbidity without affecting surgical efficacy. Comparing minimally invasive sacral colpopexy (MISC) to open SCP highlights that the minimally invasive route has lower rates of 30-day complication rates, deep vein thrombosis/pulmonary embolism, surgical site infection, readmission rates, and shorter hospital stay [3].

Another significant milestone in SCP procedure evolution was the shift from inpatient stay to outpatient settings. Previously, patients spent four days in the hospital (interquartile range (IQR) 3-5) after open SCP compared to two days after laparoscopic procedures (IQR 2-3) [4]. Despite the rising incidence of obesity causing increased operation time, MISC is still associated with shorter hospital stay [5]. Kisby CK et al. demonstrated that same-day discharge for robotic-assisted SCP is safe. No differences were observed in unplanned clinic visits, emergency department visits or readmissions [6]. Patient satisfaction appears equivalent between those discharged on the day of surgery and those who stayed the night in the hospital [7].

A 2016 review of surgery for POP found that SCP was the preferred method for apical vaginal prolapse, with a predominance of laparoscopic approaches and use of monofilament polypropylene mesh. However, the review concluded that variations exist in a majority of the technical aspects of the procedure [8]. Currently, there are 54 Obstetrics & Gynecology (OBGYN) based Female Pelvic Medicine and Reconstructive Surgery (FPMRS) fellowships and 15 Urology-based FPMRS fellowships [9]. The total number of fellowship-trained urogynecologists in the USA is 1, 378. However, there remains no definitive surgical approach or technique for performing MISC. The aim of this study was to define the tools and techniques used for various MISC steps among American Urogynecologic Society (AUGS) conference attendees and whether there are variations by age, gender, region, training background, practice setting or experience level.

## Materials and methods

A 24-item questionnaire was created about MISC (refer Appendices). We collected basic demographic information: gender, degree, years of practice, practice setting, and geographical location. If the surgeon performed MISC, we asked specific surgical questions: laparoscopic vs robotic approach, number and location of placed ports, degree and size of scope used, type and amount of suture used for vaginal and sacral attachment of the mesh, ureteral identification techniques, concomitant procedures, and timeline for hospital discharge. 

This questionnaire was reviewed/revised by the AUGS program committee for content and was approved by our local IRB. The questionnaire was given to attendees at the 2018 AUGS annual meeting in their registration packet. Participants were instructed to return the anonymous survey to the conference registration desk or via email or U.S. mail. Conference attendees were reminded on the first day of the conference to return the survey but were not otherwise contacted to increase the response rate. Survey responses were analyzed with descriptive statistics using Statistical Package for the Social Sciences (SPSS) (IBM Corp, Armonk, NY). Comparisons made between physicians of different ages, genders, years of experience, practice setting, and U.S. region were analyzed using t-tests, Fisher’s exact, or Chi-square where appropriate.

## Results

Of the 671 physician conference attendees, there were 59 responses (8.8%). Respondents were distributed across the four regions of the US: Northeast 32.1%, South 30.2%, Midwest 20.8%, and West 17%. The vast majority (94.6%) of the responders were U.S. based, with two participants from Canada and one from China. Most of the respondents were males (64.4%). Training, practice settings, and years in practice of participants are presented in Table 1.

**Table 1:** Demographic OBGYN: Obstetrics & Gynecology, FPMRS: Female Pelvic Medicine and Reconstructive Surgery

Gender	N	%
Female	21	35.6
Male	38	64.4
Degree		
OBGYN generalist	2	3.4
OBGYN FPMRS	54	91.5
Urology FPMRS	3	5.1
Practice settings		
University based	39	66.1
Community based	5	8.5
Private practice	12	20.3
Other	3	5.1
Years in practice		
Still in residency/fellowship	5	8.5
0-5 years	8	13.6
6-10 years	9	15.3
11-15 years	9	15.3
>15 years	28	47.5
Region of practice		
North East	17	30.4
Midwest	11	19.6
South	16	28.6
West	9	16.1
Outside USA	3	5.4

MISC was performed by 84.7%. Among these minimally invasive surgeons, 53.8% identified themselves as primarily robotic and 42.2% primarily laparoscopic surgeons. The remaining respondents (4.0%) performed MISC but performed the majority of cases via open approach and did not complete all parts of the survey as the questions were not applicable to their practice. 

Surgeons performed MISC through either 3 or 4 ports (50% each). Respondents primarily used a 0 degree laparoscope scope (46%) or combination of 0 and 30 degree (36%). The number of sutures used to attach mesh to the vagina varied with half of surgeons using 5-6 sutures on both the posterior (50%) and anterior (48.1%) vagina. Most responders (83.1%) used suture to attach mesh to the sacrum opposed to a tacking device. Other surgical technique responses are included in Table 2.

**Table 2:** Surgical techniques SCP: sacral colpopexy, PVR: post void residual, IV: intravenous, POD: post operative day

Mean Percentage of Procedures performed by each (%)	N	%
Robotic	N/A	53.8
Laparoscopic	N/A	42.2
Open	N/A	4.0
Amount of ports placed		
3	25	50
4	25	50
Size of scope used		
≤5	13	25.5
6-9	24	47.1
≥10	12	23.5
Scope angle most commonly used		
0 degree	23	46
30 degree	9	18
Combination of 0 and 30 degree	18	36
Do you place stents with SCP?		
yes	1	1.7
no	58	98.3
Amount of sutures most often placed on posterior vagina		
3-4	2	3.7
5-6	27	50
7-8	17	31.5
>8	6	11.1
Other	2	3.7
Amount of sutures most often placed on anterior vagina		
3-4	3	5.6
5-6	26	48.1
7-8	19	35.2
>8	4	7.4
Other	2	3.7
Do you suture to the sacrum?		
yes	49	83.1
no	10	16.9
Do you use tacks to attach to the sacrum?		
yes	5	8.5
no	54	91.5
Type of suture used to attach to sacrum		
Gortex	27	56.3
Prolene	9	18.8
Ticron	1	2.1
Ethibond	8	16.7
PDS	3	6.3
Type of tacking device used to attach to sacrum		
Protack™ (Covidien Surgical, Mansfield, MA, USA),	3	60
Did not specify	2	40
What do you use to retract large bowel		
Nothing	29	50
Suture	11	19
T-lift	6	10.3
Laparoscopic instrument	5	8.6
Robotic arm	7	12.1
How do you identify ureters on cystoscopy?		
Oral urine discoloring agent (Azo, Uribel, or Pyridium)	15	27.8
Dextrose cystoscopy fluid	1	1.9
IV methylene blue	2	3.7
IV indigo carmine	5	9.3
IV Fluorescein	13	24.1
No additional interventions	17	31.5
Do not routinely perform cystoscopy	1	1.9
How do you assess PVR post op?		
Retrograde fill the bladder, void, calculated PVR	40	75.5
Passive fill, void, bladder scan to check PVR	12	22.6
Passive fill, void, catheterize to check PVR	1	1.9
Mean percentage of patients discharged at following times (%)		
Day of surgery	n/a	15.2
POD 1 in AM	n/a	47.7
POD1 in PM	n/a	29.2
POD 2	n/a	6.1
POD3 or later	n/a	1.9

Surgeons commonly perform concomitant procedures at the time of SCP (Table 3). Midurethral sling placement was frequently performed defined as responses of “yes, often” (42.6%) or “sometimes” (51.9%). Posterior repair and perineorrhaphy were performed either “yes, often” or “sometimes” by 70.3% and 77.7%, respectively. Conversely, concomitant anterior repair was rarely performed with responses of “not usually” or “not at all” in 83.4%. Hysteropexy is rare, with 86.6% performing “not usually” or “not at all”. 

**Table 3:** Concomitant procedures Note: Values expressed as N(%)

Response	Midurethral sling	Posterior repair	Perineorrhaphy	Anterior repair	Hysteropexy
Yes, all the time	1(1.9)	0 (0.0)	0 (0.0)	0 (0.0)	3 (5.8)
Yes, often	23 (42.6)	16 (29.6)	24 (44.4)	1 (1.9)	1 (1.9)
Sometimes	28 (51.9)	22 (40.7)	18 (33.3)	8 (14.8)	3 (5.8)
Not usually	2 (3.7)	15 (27.8)	7 (13.0)	30 (55.6)	16 (30.8)
Not at all	0 (0.0)	1 (1.9)	5 (9.3)	15 (27.8)	29 (55.8)

There was more variation in physician assessment of the bladder (Table 2). All responders performed cystoscopy during MISC but used different additional means to enhance the visibility of ureteral jets. The most common responses were “no additional intervention used to identify the ureters” (31.5%), “urine discoloring medications such as Azo, Uribel, Pyridium” (27.8%), and “fluorescein” (24.1%). Post-operatively 89.8% of the surgeons assessed post void residual (PVR), most commonly via “retrograde fill followed by active voiding trial” (75.5%).

A majority of patients were discharged on post-operative day 1 (POD1) with 47.6% of patients discharged in the morning of POD1 and 29.1% discharged in the afternoon of POD1 (Table 2). Only 15.2% of patients were discharged on the day of the procedure. One participant from China reported typical discharge on POD5.

For comparisons between physicians of different ages, genders, years of experience, practice setting, and U.S. region, we only found a difference in the frequency of hysteropexy by gender. Men performed hysteropexy with SCP either “not usually” or “not at all” 97% of the time. On the contrary, women performed hysteropexy with SCP “yes, all the time”, “yes, often”, or “yes, sometimes” 33.4% of the time (p=0.03). For all other comparisons of technique there were no differences by these comparative factors. 

## Discussion

The majority of AUGS attendee survey respondents perform MISC, with an equal distribution of both laparoscopic and robotic surgeons. Concomitant posterior colporrhaphy was far more common than anterior colporrhaphy. Hystereopexy was performed more commonly by female surgeons. Beyond this variation, there is relative homogeneity of surgical technique across regions, practice settings, gender, age, and years of experience.

We found that 84.7% of responders perform sacrocolpopexy via a minimally invasive approach, and techniques are mostly homogeneous across survey respondents. We hypothesized prior to completing this research that older physicians further out from training may be less likely to use minimally invasive routes. We did not find this result, which shows that minimally invasive techniques have been relatively well adopted across AUGS. 

One of the interesting findings of this study was that while hysteropexy is less commonly performed overall, it is more commonly performed by female surgeons. Hysteropexy is an evolving technique with increasing popularity in practice. Meriwether et al. showed that laparoscopic sacral hysteropexy improves point C and vaginal length while reducing mesh exposure, without increasing blood loss or pain [10]. Currently, there is largely only short-term data on prolapse outcomes for minimally invasive hysteropexy, but with increasing use of hysteropexy there is opportunity for future research. 

Notably, this study highlights current discharge practices amongst respondents. Historically, abdominal sacrocolpopexy was associated with longer hospital stay [11]. Since the majority of the surgeons have moved towards minimally invasive approaches, time spent in the hospital after surgery has decreased. In addition, concurrently evolving Enhanced Recovery After Surgery (ERAS) protocols have further changed discharge practices. Carter-Brooks et al. demonstrated that ERAS in urogynecological populations resulted in a greater percentage of same-day discharges. Their study also showed a 93.5% patient satisfaction rate for overall surgical experience, but the authors note a slightly increased 30-day hospital readmission rate [12]. Our survey suggest that the majority of patients are discharged POD1 (76.9%), but with continued integration of minimally invasive techniques and ERAS protocols, there is potential for same-day discharge to become more routine. 

## Conclusions

In summary, our survey showed that the majority of survey respondents perform MISC split equally between robotic and laparoscopic routes. Among concomitant procedures, posterior colporrhaphy/perineorrhaphy and midurethral sling are the most common. Hysteropexies are more often performed by female surgeons. The vast majority of patients are discharged on POD1.

## Appendices

Appendix 1:

Sacral Colpopexy Questionnaire 

1.     Do you perform sacral colpopexies? 

1.     Yes, please continue with the survey

No, your survey is complete, please return it. 

2. What is your degree? 

1.     Physical therapist, your survey is complete, please return it. 

2.     Nurse practitioner, your survey is complete, please return it. 

3.     Physician assistant, your survey is complete, please return it. 

4.     Physician: OBGYN generalist 

5.     Physician: OBGYN FPMRS 

6.     Physician: Urology FPMRS 

7.     Physician: OBGYN MIGS 

8.     Other, please specify _________________________ 

3. In what setting do you practice? 

University based 

Community based 

Private practice 

Other, please specify _________________________ 

4. In what state (or country if outside the United States) are you practicing? 

5.     What is your age? ___________________ 

6.     What is your gender? 

Female 

Male 

Prefer not to disclose 

7.     How many years have you been in practice? 

Still in residency/fellowship, your survey is complete, please return it. 

0-5 years 

6-10 years 

11-15 years 

more than 15 

8.     Do you usually place ureteral stents with sacral colpopexies? 

                      a. Yes. Approximately what percentage of the time? _________ 

                      b. No 

9. What is the most common way you identify ureters cystoscopically at the end of the case: 

urine discoloring medications such AZO, Uribel, pyridium. 

10% dextrose cysto fluid 

IV Methylene blue 

IV indigo carmine 

No additional intervention used to identify ureters 

I do not routinely cysto after case 

Other (please list) _______________________ 

10.  How many sutures do you usually place to attach mesh to the posterior vagina? ___________________________________________________ 

11.  How many sutures do you usually place to attach mesh to the anterior vagina? _____________________________________________________ 

12.  How do you attach mesh to the sacrum

Suture, please list typical suture used: __________________ 

Tacking device, please list typical device used: ____________ 

Other method, please list method used: __________________ 

13.  Do you assess the post-void residual postoperatively, and if yes - how do you typically do it? 

Retrograde fill followed by voiding trial 

Remove Foley and allow bladder to fill then check PVR via bladder scan 

Remove Foley and allow bladder to fill then check PVR via catheter 

Clamp Foley and remove once urge to urinate and check a PVR 

Do not check PVR 

14.  What percent of your patients go home at the following times (please make total 100%) 

Same day as surgery _______% 

Morning of POD1 __________% 

Afternoon/Evening of POD1 ________% 

POD2 _______% 

POD#3 or later _______% 

15.  Do you perform midurethral sling with sacral colpopexy? 

Yes, all the time 

Yes, often 

Sometimes 

Not usually 

Not at all 

16.  Do you perform anterior and/or posterior repair with sacral colpopexy? 

Yes, all the time 

Yes, often 

Sometimes 

Not usually 

Not at all 

17.  Do you perform perineorrhaphy with sacral colpopexy? 

Yes, all the time 

Yes, often 

Sometimes 

Not usually 

Not at all 

18.  If uterus is still present, do you leave uterus in situ and perform sacral hystereopexy? 

Yes, all the time 

Yes, often 

Sometimes 

Not usually 

Not at all 

19.  Do you perform minimally invasive sacral colpopexies (laparoscopic or robotic approach)? 

Yes, please continue with the survey on question 29 

No, your survey is complete, please return it. 

20.  What percent of your sacral colpopexies are completed by the following routes (should sum to 100%) 

Minimally invasive (laparoscopic or robotic) __________ 

Open __________ 

21.  How many ports do you typically use? a. Two b. Three c. Four d. Five e. Six 

22.  Do you use suture or other means to retract the large bowel out of the way for sacral dissection and other parts of the procedure? 

a. Yes. Please specify ______________________ 

b. No 

23.  What size of scope do you usually use 

5mm 

10mm 

Other, please list: _______________________ 

24.  What angle of scope do you usually use 

0 degree 

30 degree 

70 degree 

Combination of the above (please list which ones): _________________________ 

Other (please list): ___________________________________________________ 
